# Around the Hospitals

**Published:** 1894-04-21

**Authors:** 


### Around the Hospitals.

[Notice to the Managers of Institutions.—Special space is now reserved for the insertion of notices of hospital meetings and festivals. The Editor requests that all notices may reach The Hospital Office, 428, Strand, at least one week before the date of each meeting.]

**The Royal Berkshire Hospital.**—No very great changes or incidents have occurred at this institution during the past year. The work has been heavier and the income smaller. The treasurer suggests the probability that the erection of cottage hospitals in the vicinity of Reading may have drawn away some of the funds. As a rule the outlying districts do not contribute a proportionate quota to the expenses of the hospital in our towns and cities, and therefore seeing that patients have increased, it would point rather to the fact that the city contributions should be larger than they now are. It is not likely that expenses will decrease. The modern demand for efficiency requires increased outlay. This is shown at Reading by the decision arrived at to appoint an extra house surgeon. It is also suggested that a dental department might be opened with advantage. This, however, is relegated to some future consideration by the Medical Committee and Governors. At present the only addition to be recorded is the room which has been furnished and placed at the disposal of the medical staff. It is situated on the ground floor of the hospital. Last year the funds of the hospital were very satisfactory; this year the handsome legacies then received are meagrely represented. From the customary collections returns have been smaller, and, indeed, from no source is an increase visible. The expenditure, on the other hand, has been larger. The Convalescent Fund also shows a balance due to the treasurer, and the only branch of the hospital work in a prosperous financial condition is the private nursing institution. The in-patients during the year numbered 1,299, and the out-patients amounted to 2,012.

**The Darlington Hospital.**—This hospital should be regarded as distinctly prosperous, since it has an income exceeding its expenditure. Nevertheless we learn that subscriptions have fallen off, this being especially the case in regard to contributions from the working classes. More prosperous times are likely to show a mending in this respect, and thus enable the hospital to maintain its efficiency and provide sufficient accommodation to the growing needs of the population. A scheme is being considered which entails the amalgamation of the Children's Hospital, maintained by Miss Pease. This would entail, it is calculated, an expenditure of £1,000 for building purposes alone, and a yearly additional expenditure of about £400. A children's ward is always an additional attraction to a hospital, but the £400 promised towards this object by Miss Pease leaves a considerable sum to be collected and expended annually.

